# Our Book Table

**Published:** 1884-03

**Authors:** 


### OUR BOOK TABLE.

A System of Oral Surgery: *being a Treatise on the Diseases and Surgery of the Mouth, Jaws, Face, Teeth, and Associate Parts;* by James E. Garretson, M. D., D. D. S. *Fourth Edition. Thoroughly Revised, with Additions.* Philadelphia: J. B. Lippincott & Co. 1884.

Immediately upon the appearance of the first edition of this work, it assumed the leading place as a text-book to which its merit and the distinguished position of its author as a surgeon and writer entitled it. The estimation in which it is held is evidenced by the demand for repeated editions, until now its fourth has been placed before the public. This is not a simple reprint of former issues. An examination of its pages shows that it has been thoroughly revised, while much new matter has been added. The third edition comprised nine hundred and sixteen pages. The fourth has ten hundred and thirty-seven, an addition of one hundred and twenty pages. Nor is it in volume alone that the improvements are visible. Many of the chapters have been almost entirely rewritten, while the order of presentation of subjects has been materially changed. So rapid is the march in dental science, that to remain in the advance, or even to keep pace with it, requires constant watchfulness on the part of authors. We are glad to see that the latest edition of this work leaves little to complain of.

"A System of Oral Surgery" gives but an imperfect idea of the comprehensiveness of the work. It might be called "A System of Dental Practice," for not only does it treat of oral surgery, but of operative and prosthetic dentistry also. It considers the qualities of the materials employed in dental practice, as well as the manner of using them, and while there is room for difference of opinion concerning methods and manipulations, few will dispute the soundness of the principles advanced.

The chapter upon Amalgams has been entirely rewritten, and large additions have been made to it. Upon the often-disputed question of the propriety of their employment, the author is explicit, but not radical. He believes most of the objections urged against it are not founded in fact, but admits that from an æsthetical standpoint it is exceptionable. He sums up the subject, however, by a generalization, which leaves the matter about where he found it.

"The place for and the use of amalgam, is to be decided by the judgment of the operator, and in proportion as such judgment is good or bad, so is a patient served or abused."

A chapter on "Inflammation," and one on "Diagnosis," have been added, and these are of exceeding great value. We have not the space or inclination to enter into a discussion concerning the pathological changes attendant upon that condition which we term inflammation, but must refer the reader to the book itself. Suffice it to say, that so much of new matter has been introduced as to make it obligatory upon every dentist, who desires to keep abreast of the advance of thought, to add the latest edition of this standard work to his library.

The publishers have issued the volume in handsome style, as, indeed, might have been predicted from their well-won reputation. The cuts are well executed, the letter-press is clear and distinct, and the binding is unexceptionable. Altogether, to say that the whole work is satisfactory, is but feebly to express the sentiments with which every intelligent dental surgeon will receive it.

DENTAL MEDICINE: *a Manual of Dental Materia Medica and Therapeutics for Practitioners and Students;* by FERDINAND J. S. GORGAS, A. M., M. D., D. D. S. Philadelphia: P. Blakeston, Son & Co. 1884.

It is an encouraging sign when repeated text-books upon materia medica and therapeutics multiply, for it indicates that dentistry has outgrown the day when it was considered but a mechanical art. Medicine is being rapidly divided into various specialties, to the great disturbance of a few old-time practitioners, but to the gratification of most thinking men, because, with the rapid growth of medical knowledge, the science has become too broad for one lifetime to suffice for the mastery of all its departments. Oral diseases are now, by intelligent medical men, relegated to the dentist. But their proper treatment requires of him a knowledge of the properties of such remedies as he will be called upon, in the proper discharge of his duties, to prescribe. The day has passed when the successful dentist could practice with but two or three medicinal agents in his case. He requires a mastery of the principles of therapeutics, and a familiarity with all the leading drugs. The publication of books like that under notice, is therefore in response to a demand, and hence is a hopeful sign of progress.

Prof. Gorgas has long been known as a ripe scholar, an eminent teacher, and a terse and vigorous writer. His long editorial and professional experience conspicuously qualifies him for the preparation of such awork as this, and a brief examination of it convinces the reader that he has not miscalculated his ability. It is a book of over three hundred pages, enriched with many tables and formularies, with a great deal of untabulated information, the results of the extended experience and studies of the author. It is something more than a materia medica, for it contains a chapter on "Diagnosis" that is well worth the price of the book. Such useful information, compressed into almost epigrammatic paragraphs, is peculiarly welcome to practitioners who have not the leisure for extended study, and the book is full of it. We most heartily commend it to every one who is desirous of a complete text-book upon dental therapeutics.

LETTERS FROM A MOTHER TO A MOTHER *on the Formation, Growth and Care of the Teeth; by the Wife of a Dentist. Mrs. M. W. J.*

This series of letters, written by the author for the *Southern Dental Journal,* has attracted wide-spread attention for the easy and graceful style in which the letters are written, no less than from their eminently practical character. They contain a mass of information which it would be well for every mother carefully to study, and their republication in pamphlet form was in response to an imperative demand. Every alternate dentist has written and published chapters upon the management and care of the teeth, but nothing from the standpoint of the mother has appeared before, and that makes this series peculiarly valuable. There would be a marked improvement in the next generation of men and women if every parent would not only procure, but carefully study this little work.

From the technical standpoint of the professional man it is open to many criticisms, but it was written for popular and not professional reading, and we are not therefore called upon to point them out. It may be procured by addressing Judson & Dunlap, Atlanta, Ga. Price, $1.50 per dozen.

ARCHIVES OF PEDIATRICS: *a Monthly Journal, devoted to the Diseases of Infants and Children. Edited by* WILLIAM PERRY WATSON, A. M., M. D. Jersey City, N. J.: $3.00 per annum.

The mere mention of the name of this new journal is sufficient to indicate that it will fill a vacant place, and that to dentists especially it will be of inestimable value, the more so when it is known that so able a writer as Dr. Watson, assisted by a brilliant corps of collaborators, will conduct it. The initial number denotes that it will be of an unusually high order. We wish it the most abundant success.

THE ANALECTIC: *A Monthly Periscopic Summary of the Progress of Medical Science. Edited by* WALTER S. WELLS, M. D., New York. G. P. Putnam's Sons. $2.50 per year.

To the busy professional man who has not the leisure to read all the principal periodicals, we can heartily commend this new journal. It is what its name indicates, a summary of all that is new and valuable in medical science; not simply excerpts, but condensations; the very pith extracted from extended articles.

